# Functional Integrity of Radical SAM Enzyme Dph1•Dph2 Requires Non-Canonical Cofactor Motifs with Tandem Cysteines

**DOI:** 10.3390/biom14040470

**Published:** 2024-04-11

**Authors:** Koray Ütkür, Klaus Mayer, Shihui Liu, Ulrich Brinkmann, Raffael Schaffrath

**Affiliations:** 1 Institut für Biologie, Fachgebiet Mikrobiologie, Universität Kassel, 34132 Kassel, Germany; k.uetkuer@uni-kassel.de; 2 Roche Pharma Research and Early Development (pRED), Large Molecule Research, Roche Innovation Center Munich, 82377 Penzberg, Germany; klaus.mayer.km1@roche.com (K.M.); ulrich.brinkmann@roche.com (U.B.); 3 Division of Infectious Diseases, Department of Medicine, University of Pittsburgh, Pittsburgh, PA 15261, USA; shl176@pitt.edu

**Keywords:** Dph1•Dph2, radical SAM enzyme, iron sulfur cluster, cysteine ligands, eEF2, diphthamide modification, ADP-ribosylation, *Saccharomyces cerevisiae*

## Abstract

The Dph1•Dph2 heterodimer from yeast is a radical SAM (RS) enzyme that generates the 3-amino-3-carboxy-propyl (ACP) precursor for diphthamide, a clinically relevant modification on eukaryotic elongation factor 2 (eEF2). ACP formation requires SAM cleavage and atypical Cys-bound Fe-S clusters in each Dph1 and Dph2 subunit. Intriguingly, the first Cys residue in each motif is found next to another ill-defined cysteine that we show is conserved across eukaryotes. As judged from structural modeling, the orientation of these tandem cysteine motifs (TCMs) suggests a candidate Fe-S cluster ligand role. Hence, we generated, by site-directed *DPH1* and *DPH2* mutagenesis, Dph1•Dph2 variants with cysteines from each TCM replaced individually or in combination by serines. Assays diagnostic for diphthamide formation in vivo reveal that while single substitutions in the TCM of Dph2 cause mild defects, double mutations almost entirely inactivate the RS enzyme. Based on enhanced Dph1 and Dph2 subunit instability in response to cycloheximide chases, the variants with Cys substitutions in their cofactor motifs are particularly prone to protein degradation. In sum, we identify a fourth functionally cooperative Cys residue within the Fe-S motif of Dph2 and show that the Cys-based cofactor binding motifs in Dph1 and Dph2 are critical for the structural integrity of the dimeric RS enzyme in vivo.

## 1. Introduction

Radical SAM (RS) enzymes contain iron-sulfur (Fe-S) cluster cofactors associated with the reductive cleavage of S-adenosyl-methionine (SAM) and the formation of classical 5′-deoxyadenosine (dAdo) radicals [1,2]. These are used for H-atom abstraction from substrates in a plethora of cellular syntheses including but not limited to modifications of biomacromolecules [1,2,3]. Classical members of the RS family share a radical SAM motif (CX_3_CX_2_C) with each cysteine coordinating one iron of the cofactor, usually a cubic [4Fe-4S] cluster. The fourth, unique iron binds SAM to set the catalytic framework of a site-differentiated Fe-S cluster for reductive SAM cleavage [3,4]. The Cys-bound Fe-S clusters not only are crucial for SAM catalysis, but also for RS enzyme structure and stability. Hence, the mutation of crucial cysteines in the SAM motif can induce Fe-S cluster loss as well as enzyme inactivation or degradation [5,6].

Apart from classical RS enzymes, there are non-canonical ones, i.e., eukaryotic Dph1•Dph2 and archaeal Dph2•Dph2 orthologs, that significantly differ with regard to subunit composition, Fe-S cluster coordination, SAM cleavage, and radical formation [7,8,9,10]. Dph1•Dph2 processes SAM into a non-canonical 3-amino-3-carboxy-propyl (ACP) radical used for the modification of eukaryotic translation elongation factor 2 (eEF2) with diphthamide [11,12,13]. Diphthamide is formed via a dedicated synthesis network (i.e., Dph1-Dph8) on a conserved histidine of eEF2 in eukaryotes (His-699 in budding yeast *Saccharomyces cerevisiae* and His-715 in *Homo sapiens*) and EF2 in some archaea [14,15,16,17]. Importantly, human patients deficient in diphthamide-modified eEF2 display symptoms of a neurodevelopmental disorder known as diphthamide deficiency syndrome (DDS) [18,19,20,21,22] and diphtheria toxin (DT) from *Corynebacterium diphtheriae* attacks the diphthamide décor on eEF2 by ADP-ribosylation to block mRNA translation in infected host cells [23]. Yeast diphthamide mutants (i.e., *dph1*Δ–*dph8*Δ) are resistant to growth inhibition by DT, which is why the toxin is a valuable molecular tool to tell diphthamide proficiency from deficiency in vivo [17,24].

The yeast Dph1•Dph2 heterodimer contains one Fe-S cluster per subunit, yet both Dph1 and Dph2 lack the consensus (CX_3_CX_2_C) typical of classical radical SAM domains [9,10]. Instead, Fe-S cluster coordination relies on atypical Cys-based motifs in Dph1 (CX_105_CX_129_C) and Dph2 (CX_21_CX_234_C), which upon mutation cause Fe-S cluster loss and diphthamide defects [9,10] and, importantly, are verified triggers of DDS [21]. Intriguingly, there are other cysteines with ill-defined roles in protein function and stability: Dph1Cys-134 and Dph2Cys-106. Both form tandem cysteine motifs (TCMs) with Cys residues previously implicated in Fe-S cluster binding: Dph1Cys-133 and Dph2Cys-107 [9,10]. As judged from sequence alignments with other eukaryotic Dph1 and Dph2 subunits and structural Dph1•Dph2 modeling, we show that the TCMs are conserved from yeast to man and possibly expand the atypical Cys-based cofactor binding motifs. Therefore, we generated *DPH1* and *DPH2* gene substitutions for the functional analysis of site-specific Cys-to-Ser variants in either Dph1 or Dph2. Assays diagnostic for diphthamide synthesis reveal that while the replacement of either Cys-106 or Cys-107 alone in Dph2 had mild effects, their combined substitution almost entirely abolished Dph1•Dph2 activity. Furthermore, we show that irrespective of position, replacing essential cysteine ligands from SAM and/or Fe-S binding motifs in Dph1 or Dph2 results in the accelerated decay of both subunits unanimously. Collectively, our data establish the relevance of TCMs and Cys-based motifs in Dph1 and Dph2 as factors that determine the function and structural integrity of the Dph1•Dph2 dimer.

## 2. Materials and Methods

### 2.1. Strains, Media, and Cell Growth Conditions

The *S*. *cerevisiae* strains used or generated throughout this study are listed in Table S1. BY4741-derived yeast strains carrying site-specific substitution or deletion mutations at *DPH1* or *DPH2* chromosomal loci were generated using PCR-mediated protocols, oligonucleotides, gene-specific primers (Table S2), and plasmid templates, as previously described [10,24,25]. HA or c-Myc epitope tagging at wild-type and mutant *DPH1* or *DPH2* loci for Dph1 or Dph2 gene product detection involved previously described PCR methods [26]. DNA transformations utilized standard lithium-acetate protocols [27], and strains were grown in complete yeast peptone dextrose (YPD) or minimal synthetic defined (SD) media [28] at 30 °C. For antifungal-response assays, ten-fold serial cell dilutions of *S. cerevisiae* tester strains (starting OD_600_: 1.5) were spotted on YPD plates lacking or containing 5–15 μg/mL sordarin (Sigma-Aldrich, Darmstadt, Germany) and incubated for 2–4 days at 30 °C. As previously described [24], DT-response assays used transformation with a plasmid (pSU9) for the galactose induction of the cytotoxic ADP-ribosylase domain of DT.

### 2.2. Dph1•Dph2 Sequence Alignments and Modeling Based on Archaeal Dph2 Structures

The sequence of *Pyrococcus horikoshii* (*Ph*) Dph2 (UniProt-ID O58832) was aligned to Dph1 and Dph2 from *S. cerevisiae* (*Sc*) (UniProt-IDs P40487 and P32461), *Arabidopsis thaliana* (*At*) (UniProt-IDs Q8RWW3 and A0A1I9LRW3), *Drosophila melanogaster* (*Dm*) (UniProt-IDs Q9VTM2 and Q9VFE9), *Mus musculus* (*Mm*) (UniProt-IDs Q5NCQ5 and Q9CR25), or *Homo sapiens* (*Hs*) (UniProt-IDs Q9BZG8 and Q9BQC3) using ClustalOmega (https://www.ebi.ac.uk/Tools/msa/clustalo/ accessed on 5 February 2024) and illustrated using Jalview (https://www.jalview.org/development/archive/Version-2_11_2_7/ accessed on 5 February 2024). AlphaFold/ColabFold [29,30]-based models of Dph1•Dph2 were guided by the solved structure of *Ph*Dph2 (PDB:3LZD) [7,12], as described in [10,21] (https://colab.research.google.com/github/sokrypton/ColabFold/blob/main/AlphaFold2.ipynb accessed on 5 February 2024). Structure visualization used PyMOL (https://pymol.informer.com/1.3/ accessed on 5 February 2024), and the cysteine residues of the atypical radical SAM and/or Fe-S binding motifs were curated manually.

### 2.3. Assaying Diphthamide Modification of eEF2 and ADP-Ribosylation (ADPR) by ETA

Diagnosis of eEF2 diphthamide-modification states in vivo involved Western blots of total yeast cell extracts with antibodies that recognize global eEF2 pools irrespective of diphthamide modification (anti-eEF2[pan]) or unmodified forms of eEF2 (anti-eEF2[no diphthamide]) [31]. Both antibodies were originally raised to detect human eEF2. As the diphthamide acceptor contexts in eEF2 from human and yeast cells are identical, anti-EF2(no diphthamide) can also differentiate the modification states of eEF2 in *S. cerevisiae* [32] and in plants, as recently shown in [33]. Unmodified eEF2 signals relative to *dph1*∆ or *dph2*∆ were quantified using densitometry with ImageJ version 1.50i. Yeast cell extracts were generated as described [34] and protein concentrations were determined using the Bradford assay [35]. Lämmli samples were run using SDS-PAGE (12% [*w*/*v*] polyacrylamide) and blotted onto PVDF membranes (Millipore, Burlington, MA, USA). These were probed overnight at 4 °C with the anti-eEF2(pan) and anti-eEF2(no diphthamide) antibodies and developed using anti-rabbit secondary antibody horseradish-peroxidase conjugates (Dianova, Hamburg, Germany; working concentration: 1/5000) and Lumi-Light Western blotting substrate (Roche, Basel, Switzerland), as described [10,21,32]. Protein loading was controlled in parallel with anti-Cdc19 antibodies (kindly donated by Prof. Jeremy Thorner, University of California, Berkeley, CA, USA), recognizing yeast pyruvate kinase. Similarly, anti-HA Western blots (Invitrogen, Waltham, MA, USA) were performed to detect HA-tagged Dph1 proteins, and anti-c-Myc Western blots (9E10 antibody kindly donated by Prof. Markus Maniak, University of Kassel, Germany) confirmed the expression of c-Myc-tagged Dph2 proteins. Cycloheximide chases followed a previously described protocol [36]. In brief, yeast was grown overnight to inoculate (OD_600_: 0.5) the main cultures for 3h until the exponential phase prior to cycloheximide [100 μg/mL] addition. Fractions of cultures were taken before (0 h) and up to nine h after cycloheximide treatment followed by Western blot analysis as described above. The diphthamide-dependent ADPR acceptor activity of eEF2 in the presence of *Pseudomonas aeruginosa* exotoxin A (ETA), an ADP-ribosylase similar to DT [23], was tested in vitro using yeast extracts and biotinylated NAD^+^ as ADP-ribosyl a donor for ETA and a streptavidin-peroxidase conjugate (Roche, Basel, Switzerland) to detect ADPR on eEF2 [24,37].

## 3. Results and Discussion

### 3.1. Tandem Cysteine Motifs (TCM) in Dph1 and Dph2 Are Conserved from Yeast to Humans

**Figure 1:**
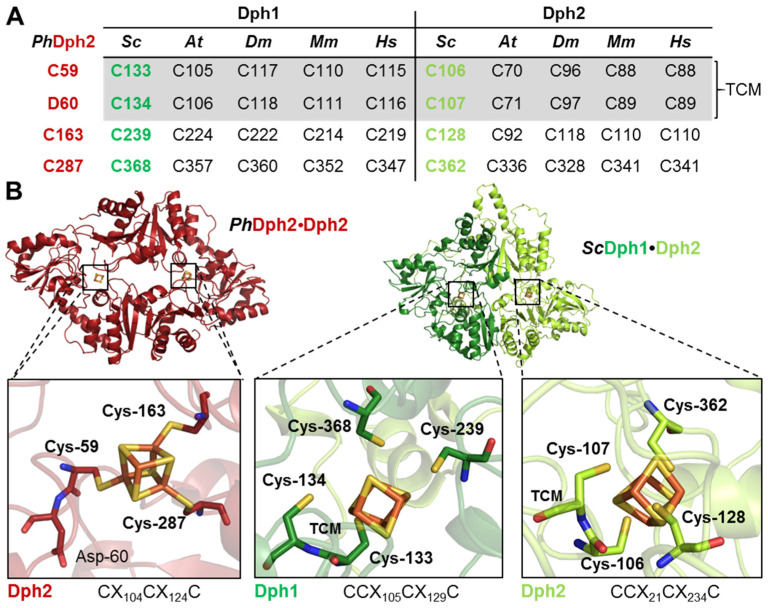
TCM conservation in atypical radical SAM and Fe-S motifs from Dph1•Dph2 dimers. (**A**) Dph1 and Dph2 alignment from indicated eukaryotic species to archaeal Dph2 (for full details, see Figure S1). (**B**) Structural comparisons. Left: structure of the *Ph*Dph2•Dph2 homodimer (firebrick red; PDB:3LZD) with the Cys-based (Cys-59; Cys-163; Cys-287) Fe-S binding motif previously established [7,9,10,12]. Note Cys-59 is next to Asp-60. Right: an AlphaFold model of the *Sc*Dph1•Dph2 heterodimer (forest and lemon green) confirming that cysteines in Dph1 (Cys-133; Cys-239; Cys-368) and Dph2 (Cys-C107; Cys-128; Cys-362) are proximal to respective Fe-S clusters. As part of TCMs, a fourth cysteine, Cys-134 next to Cys-133 in Dph1 and Cys-106 next to Cys-107 in Dph2, orients towards each Fe-S cluster. Fe-S motif close-ups are 66% transparent for emphasis on stick structures. Dph1•Dph2 was modelled and illustrated as previously described [10,21].

Atypical cofactor binding motifs from Dph1•Dph2 have been identified elegantly in *S. cerevisiae* based on sequence conservations between its archaeal Dph2•Dph2 ortholog from *P. horikoshii* (*Ph*), whose crystal structure has been solved [7,9,12]. Intriguingly, Dph1 and Dph2 sequences from yeast (but not *Ph*Dph2) revealed that the first Fe-S-cluster binding cysteine is next to another one of unknown relevance. Thus, in Dph1, uncharacterized Cys-134 is in tandem with Fe-S-cluster ligand Cys-133, and a similar scenario applies to Dph2 with a ligand Cys-106 adjacent to Cys-107 [9,10]. Since their significance is unclear, we examined whether TCMs are unique to yeast or found in other eukaryotes and aligned Dph1 and Dph2 sequences between *S. cerevisiae* (*Sc*), *A. thaliana* (*At*), *D. melanogaster* (*Dm*), *M. musculus* (*Mm*), and *H. sapiens* (*Hs*) (Figure S1). Compared to *Ph*Dph2, which displays a Cys-Asp motif, all eukaryal Dph1 and Dph2 sequences tested carry a Cys residue in position 1 of the Fe-S-cluster motif that indeed forms a conserved TCM (Figure 1A). We modeled the yeast Dph1•Dph2 dimer with AlphaFold using the solved structure of *Ph*Dph2 to further characterize the cryptic Cys residues in each TCM (Figure 1B). The model identifies both verified and potential Fe-S-cluster ligands [9] in Dph1 (Cys-133, Cys-239, and Cys-368) and Dph2 (Cys-107, Cys-128, and Cys-362) (Figure 1B, middle and right panels), in line with those from *Ph*Dph2 (Cys-59, Cys-163, and Cys-287) (Figure 1B, left panel). Strikingly, the cryptic cysteines in each TCM (i.e., Dph1Cys-134 and Dph2Cys-106) also are oriented towards the Fe-S cofactors, suggesting they may qualify as candidate ligands (Figure 1B, middle and right panels). In contrast, Asp-60 next to Fe-S cluster ligand Cys-59 in *Ph*Dph2, faces away from the cofactor (Figure 1B, left panel). In sum, we surmised that four rather than three cysteines may be atypical cofactor motifs in Dph1 (CCX_105_CX_129_C) and Dph2 (CCX_21_CX_234_C) (Figure 1B) and function as candidate Fe-S ligands.

### 3.2. Diphthamide-Relevant Cooperation of Cys-106 and Cys-107 in the TCM of Dph2

**Figure 2:**
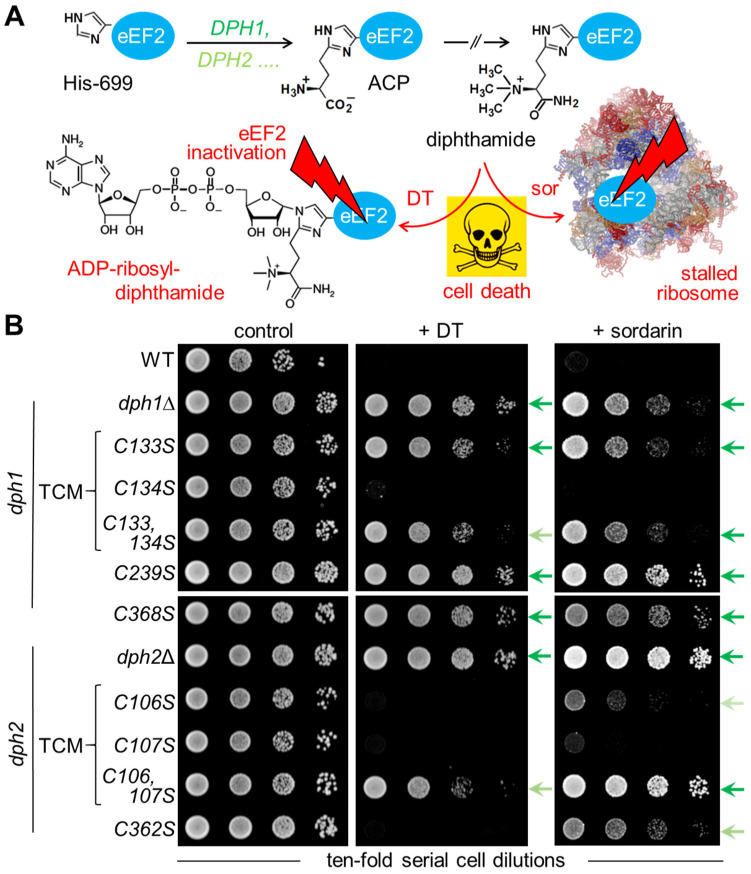
*DPH1* and *DPH2* mutagenesis to study TCM relevance for Dph1•Dph2 activity in vivo. (**A**) Simplified diphthamide pathway with *DPH1* and *DPH2* genes required to initiate ACP formation [21]; subsequent *DPH* products and steps for the completion of diphthamide synthesis [14,15,16,17] are not detailed. Diphthamide can be hijacked to induce cell death (skull–crossbones) either by DT for lethal ADP-ribosylation of eEF2 or sordarin (sor), which in a complex with the décor on eEF2 stalls ribosomes [40]. (**B**) Growth assays in response to DT and sordarin for the diagnosis of diphthamide defects in vivo. Yeast tester strains comprised wild-type (WT: *DPH1 DPH2*) and null mutants (*dph1*∆; *dph2*∆) together with single/double *DPH1* or *DPH2* gene substitutions, as indicated. Cells were grown w/o DT and sordarin (control: left panel), under DT-inducing conditions (+ DT: middle panel) [24], or with sordarin (+ sordarin: right panel) doses sufficient to inhibit WT cells. Arrows shaded in green denote various degrees of DT and sordarin resistance.

Next, we examined the relevance, if any, of the TCMs (i.e., Dph1Cys-133 and Cys-134; Dph2Cys-106 and Cys-107) for diphthamide synthesis on eEF2 in vivo. We generated site-directed Cys-to-Ser-substitution variants of Dph1•Dph2 containing individual and combined TCM replacements in subunits Dph1 (*C133S*, *C134S*, *C133*,*134S*) and Dph2 (*C106S*, *C107S*, *C106*,*107S*). For comparison, we included null mutants (*dph1*Δ; *dph2*Δ) and Dph1•Dph2 variants (*dph1C239S*; *dph1*C*368S*; *dph2C362S*) with verified or suspected Fe-S ligand defects [9,10]. Next, the diphthamide synthesis capacity was investigated in vivo with assays monitoring diphthamide-dependent cell growth inhibition by DT and sordarin (Figure 2A). The latter antifungal stalls ribosomes and blocks protein synthesis in a fashion unrelated to DT but also dependent on the diphthamide décor on eEF2 [38,39,40].

First, tester strains were transformed with pSU9, a plasmid allowing for the expression of the catalytic DT subunit under *GAL1* promoter control [24]. Under inducing conditions, i.e., with galactose added to the growth medium as a sole carbon source (Figure 2B), a sensitive DT phenotype leading to cell death was seen with diphthamide-proficient wild-type (WT) cells (Figure 2B). In contrast, DT-resistance traits comparable to diphthamide-deficient *dph1*Δ and *dph2*Δ null controls were found to be triggered by Cys-to-Ser substitution in the *DPH1* (*C133S*, *C133*,*134S*, *C239S*, *C386S*) and *DPH2* (*C106*,*107S*) genes (Figure 2B). Intriguingly, compared to the latter double mutant, which has both cysteines in the TCM of Dph2 replaced by serines (*C106*,*107S*), the substitution of each cysteine alone (*C106S* or *C107S*) did not protect either single mutant against DT (Figure 2B). This is a phenotypic read-out indicative of a functional overlap between Cys-106 and Cys-107 in the TCM of Dph2. Consistently, functional cooperation among the two cysteines can also be deduced from (DT-independent) assays that monitor resistance towards the diphthamide indicator antifungal sordarin [38,39,40]. Here, phenotypic additivity between Cys-106 and Cys-107 is even more pronounced (compared to the DT assay), and the double-substitution mutant (*C106*,*107S*) conferred sordarin resistance as robust as the *dph2*Δ null control, lacking Dph1•Dph2 activity altogether (Figure 2B). This outcome significantly differs from the phenotypes triggered by the respective TCM substitutions in Dph1 (Figure 2B). While one of the single mutants (*C133S*) copies the DT and sordarin resistance traits of the double mutant (*C133*,*134S*), the other single mutant (*C134S*) displayed WT-like sensitivities to either of the diphthamide-indicator agents (Figure 2B). Thus, in the TCM of Dph1, Cys-133 apparently is the major catalytic driver, and Cys-134 plays no such role for diphthamide synthesis by the RS enzyme.

As with previously reported assays [10,24,32,39,41], we noticed that both DT and sordarin triggered phenocopies in most of our genetic backgrounds (Figure 2B), which is in further support of their use as bona fide diphthamide indicators. However, in case of the *DPH2* substitution (*C362S*), which, when mutated together with Cys-107 (*C107*,*362S*), was reported to have an Fe-S cluster defect in vitro [9], we observed an exception from this principle, i.e., the separation of DT sensitivity from sordarin resistance (Figure 2B). Whether such phenotypic heterogeneity is unique to the mutant (*C362S*) and reflects a specific difference in response to both cytotoxic agents, which, in spite of sharing the requirement for diphthamide, have distinct modi operandi [23,40], is not known to the best of our knowledge. Nonetheless, it is feasible to this end that eEF2 diphthamide modification states sufficient to undergo lethal ADPR via DT (i.e., sensitivity phenotype) may not be enough to be targetable by sordarin and freeze eEF2 on the ribosome to kill yeast cells (i.e., resistance-trait phenotype) [23,40].

### 3.3. Cys Substitutions in the SAM and Fe-S Motifs Trigger Unmodified eEF2 Pools

**Figure 3:**
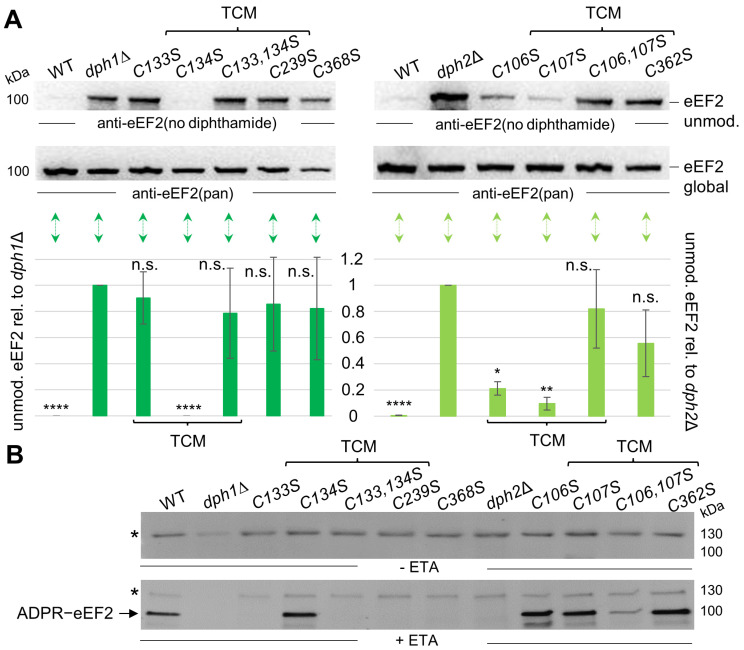
Accumulation of unmodified eEF2 in Cys-to-Ser Dph1•Dph2 mutants reveals functional roles of TCMs in diphthamide synthesis. (**A**) Western blots diagnostic for diphthamide synthesis on eEF2 from cell extracts of indicated yeast strains; anti-eEF2(no diphthamide) antibodies specific for unmodified eEF2 (upper panels) produce immune signals that associate with dysfunctional Dph1•Dph2 rather than WT enzymes, and anti-eEF2(pan) antibodies (middle panels) recognize eEF2 irrespective of modification state [31,32,33]. Technical repetitions (n = 3) were quantified (lower panels) using t-test statistics (* = *p* < 0.05; ** = *p* < 0.01; **** = *p* < 0.0001; n.s. = not significant). (**B**) ADP-ribosylation (ADPR) assay. Cell extracts incubated w/o (upper panel) or with exotoxin A (ETA) (500 ng, lower panel) with biotin-NAD^+^ allowed for diphthamide-dependent biotin-ADP-ribose transfer (ADPR-eEF2). Detection was performed via Western blot using a streptavidin-HPR-conjugate. Asterisks mark an unspecific ADPR band previously described [24]. Original images can be found in the Supplementary Materials (Figures S2–S4).

Next, we analyzed the Cys-to-Ser variants using Western blots with anti-eEF2(no diphthamide) antibodies that specifically recognize unmodified eEF2 [31,32,33] (Figure 3A). Thus, in support of our phenotypic assays above (Figure 2B), such immune blots can provide further insights into the relevance of each cysteine replaced in our Dph1 or Dph2 substitution variants and confirm that modified eEF2 samples from WT cells with active Dph1•Dph2 enzymes will not respond towards this diagnostic antibody [31,32,33]. While our WT control contained next to no unmodified eEF2, the *dph1*∆ and *dph2*∆ deletion strains accumulated substantial pools of eEF2 not modified by diphthamide (Figure 3A). Based on this rationale, we detected pools of unmodified eEF2 in *DPH1* single *C133S*, *C239S*, and *C368S*, as well as double *C133*,*134S* mutants that compared to *dph1*∆ signals (Figure 3A, left panel). In contrast, eEF2 diphthamide-modification states in the *DPH1* single-substitution mutant *C134S* resembled WT pools bare of any unmodified eEF2 (Figure 3A, left panel). We conclude that in contrast to the robust defect seen with the *dph1C133S* mutant, the *C134S* substitution is fully proficient in diphthamide synthesis. This read-out, which complements our data from the DT and sordarin assays above (Figure 2B), reconfirms that within the TCM of Dph1, Cys-133 (not Cys-134) is the major diphthamide driver.

In relation to *DPH1*, the set of cysteine mutations in the *DPH2* gene uncovered a more complex functional profile (Figure 3A, right panel) that goes hand-in-hand with the in vivo phenotypes above (Figure 2B). Again, individual replacements of Cys-106 (*C106S*) and Cys-107 (*C107S*) within the TCM of Dph2 produced small pools of unmodified eEF2, yet to a significantly lesser degree than the *dph2*∆ null control (Figure 3A right panel). In contrast, the double mutant (*C106*,*107S*), lacking the TCM in Dph2 altogether, triggered substantial amounts of unmodified eEF2 (Figure 3A right panel). In fact, the diphthamide defect seen for double-mutant (*C106*,*107S*) cells compares to unmodified eEF2 pools from *dph2*∆ nulls, and the Fe-S ligand mutant (*C362S*) [9] (Figure 3A, right panel), which we showed above, displays phenotypic heterogeneity (Figure 2B).

We next aimed to verify the Western blot data above using an independent assay in vitro [37] (Figure 3B). It is based on ETA, which, similarly to DT, requires the diphthamide décor to attack eEF2 by ADP-ribosylation in a reaction involving NAD^+^ as an ADP-ribose donor (Figure 3B). In the assay, the use of biotinylated NAD^+^ enabled us to monitor the ADPR modification on eEF2 through streptavidin-based Western blots [37]. Functionally compromised or inactive Dph1•Dph2 renders eEF2 unmodified and hence less sensitive or resistant to the ADPR attack by ETA, resulting in reduced or a lack of Western signals in relation to WT cells with an active Dph1•Dph2 enzyme (Figure 3A). While diphthamide-deficient *DPH1* mutants *C133S*, *C239S*, and *C368S* displayed no ADPR acceptor band for eEF2, the *C134S* mutant showed WT-like ADPR patterns (Figure 3B). This finding, which is in line with the WT-like read-outs from the other assays above (Figure 2B and Figure 3A), supports the view that Cys-134 is functionally dispensable from the TCM in Dph1. Markedly, ADPR signals on eEF2 observed in particular from *DPH2* mutants (*C106*,*107S* and *C362S*) point towards detectable diphthamide levels (Figure 3B), even though in vivo phenotypes (Figure 2B) and anti-eEF2 Western blots implied diphthamide defects (Figure 3A). In spite of Cys-106 and Cys-362 being verified as Fe-S-cluster ligands in Dph2 that lose cooperativity when mutated in tandem (*C107*,*362S*) [9], we can thus conclude that Dph1•Dph2 activities in each of our single mutants (*C107S* or *C362S*) are decreased, not abolished. Thus, residual diphthamide levels produced from each mutant background may be sufficient enough to generate the observed pools of ADPR-eEF2. Such a scenario is not unheard of and was reported before with regard to a subset of pathogenic and clinically important variants of DPH1•DPH2 from human DDS patients [10,19,20,21].

Strikingly, in relation to each single mutant (*C106S* or *C107S*) alone, eEF2 from the *DPH2* double substitution (*C106*,*107S*) variant displayed a drastic decrease in ADPR acceptor activity (Figure 3B). Again, this finding indicates a robust reduction in RS enzyme activity when both Cys residues of the TCM in Dph2 have been replaced by Ser residues, (Figure 3B) and goes hand-in-hand with our data from the Western blots above, which demonstrate that Cys-106 and Cys-107 cooperate with one another and confer full functionality to the Dph1•Dph2 heterodimer. In sum, our TCM analysis in Dph1 demonstrates that while Cys-134 clearly is dispensable, Cys-133 is essential for Dph1•Dph2 enzyme activity and diphthamide synthesis on eEF2. Thus, together with previous studies on the atypical radical SAM domain in Dph1 [9,10], Cys-133 appears to be critical for Fe-S-cluster coordination. As for Dph2, our TCM analysis and diphthamide profiles uncover that the replacement of Cys-106 or Cys-107 alone results in a partial loss of enzyme activity, while a lack of both dramatically compromises Dph1•Dph2 function. Therefore, we propose a cooperative role between Cys-106 and Cys-107 in the TCM of Dph2 that likely supports Fe-S-cluster binding and Dph1•Dph2 enzyme function.

### 3.4. Mutations in the SAM and Fe-S Motifs Drastically Decrease Dph1•Dph2 Amounts

**Figure 4:**
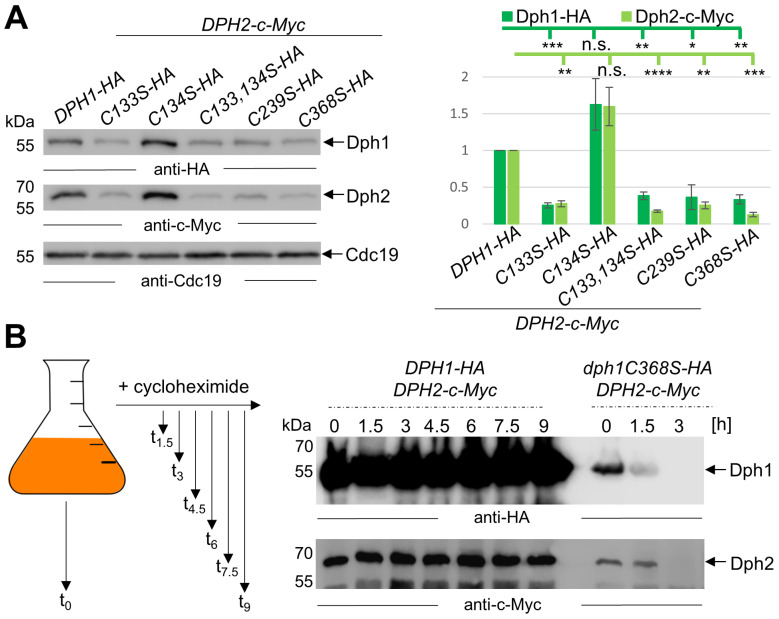
Malfunctional Cys-to-Ser variants of the Dph1•Dph2 dimer have significantly decreased amounts of Dph1 and Dph2 subunits. (**A**) Western blot detection of Dph1-HA (anti-HA) and Dph2-c-Myc (anti-c-Myc) from total extracts of indicated *DPH1-HA* substitution mutants co-expressing *DPH2-c-Myc* (left panel). Detection of Cdc19 (anti-Cdc19) served as internal control. Technical repetitions (n = 3) were followed by densitometric quantification of signal intensities (right panel) and t-test statistics (* = *p* < 0.05; ** = *p* < 0.01; *** = *p* < 0.001; **** = *p* < 0.0001; n.s. = not significant). (**B**) Cycloheximide chase of *dph1C368S* reveals accelerated decay of Dph1•Dph2. Schematic workflow of the cycloheximide chase (left panel). Yeast cells coding for HA and c-Myc-tagged Dph1 and Dph2 were grown to the exponential phase (t_0_) before the addition of cycloheximide. Samples were taken at indicated time points before (t_0_) and after cycloheximide addition for protein extraction and Western blot analysis (right panel). Original images can be found in the Supplementary Materials (Figures S5 and S8).

Previously, yeast Dph1•Dph2 variants with Cys-to-Ala substitutions were reported with low protein yields when produced from recombinant bacteria [9]. In line with this, we reported that the *DPH1*-substitution mutant (*C368S*) produced significantly lower Dph1 amounts than WT cells [10]. Hence, we studied Dph1•Dph2 levels in our collection of mutants with HA and c-Myc epitope-tagged versions of the Cys-to-Ser variants generated by PCR-mediated protocols in vivo. Yeast strains co-expressing Dph1-HA variants with Dph2-c-Myc (Figure 4A and Figure S5) or Dph2-c-Myc variants with Dph1-HA (Figures S6 and S7) were analyzed via Western blots using anti-HA and anti-c-Myc antibodies to detect each subunit in the Dph1•Dph2 populations. As for the *DPH1* set of mutations, cellular amounts of all HA-tagged Cys-to-Ser variants (*C133S*; *C133,134S*; *C239S*; *C368S*)—except for the one (*C134S*) with WT-like properties based on the assays above (Figure 2 and Figure 3)—were significantly decreased (Figure 4A, left panel). In fact, Dph1-HA levels in all these mutants (*C133S*; *C133*,*134S*; *C239S*; *C368S*) had dropped to 25–38% (Figure 4A, right panel) of the WT control (*DPH1-HA DPH2-c-Myc*). Strikingly, we also observed a drastic decrease in their amounts of Dph2-c-Myc (Figure 4A, left panel). Thus, albeit encoded from otherwise native genomic *DPH2* loci, the Dph2-c-Myc levels had dropped to 13–27% of WT levels (Figure 4A, right panel) in all *dph1* mutants (*C133S C133*,*134S*; *C239S*; and *C368S*). Again, the active (*C134S*) mutant deviated from this pattern. producing proper or even higher WT pools of the epitope-tagged Dph1•Dph2 dimer (Figure 4A). Thus, dramatically reduced Dph1 and Dph2 protein levels occur in the very Cys-Ser substitutions of the *DPH1* gene product that interfere with the radical SAM motif in Dph1 and compromise diphthamide synthesis [9,10,42].

Similarly, we observed a reduction of Dph1•Dph2 enzyme and subunit levels with the *DPH2* set of mutations (Figures S6 and S7), albeit not as severe as with the *dph1* mutant collection above (Figure 4A). Among *dph2* mutants tested (*C106S*; *C107S*; *C106*,*107S*; and *C362S*), the levels of Dph2-c-Myc and Dph1-HA dropped to, respectively, 40–71% and 30–56% relative to the WT (Figures S6 and S7). *dph2* mutants found to be severely compromised (*C106*,*107S*; *C362S*) in Dph1•Dph2 activity on the basis of phenotypic (Figure 2B), anti-eEF2 (Figure 3A), and ADPR (Figure 3B) assays maintained Dph2-c-Myc or Dph1-HA at reduced but significantly higher levels compared to the most affected *dph1* counterparts (*C133S*; *C133*,*134S*; *C239S*; and *C368S*) (Figure 4A). This suggests that the loss of the capacity to synthesize diphthamide in each of the mutant Dph1•Dph2 populations may not be solely ascribed to changed levels in Dph1 and/or Dph2 subunits. In support, we observe significantly lesser Dph1•Dph2 instability in the inactive *dph2C362S-c-Myc DPH1-HA* mutant (Figures S6 and S7), which according to Dong et al. (2019) is malfunctional due to a binding defect of a regulatory rather than catalytic Fe-S-cluster Dph2 [9].

### 3.5. Non-Canocical SAM Motifs Ensure Dph1•Dph2 Stability in Yeast Cells over Time

To further address Dph1•Dph2 instability as a direct or indirect result from Cys-to-Ser substitutions in the *DPH1* or *DPH2* gene products (Figure 4A), we chose a cycloheximide chase experiment [36] followed by Western blots (Figure 4B). Cycloheximide inhibits translation in eukaryotes in a fashion involving competition with the acceptor end of deacetylated tRNA in the ribosome [43,44]. Eventually, translation becomes stalled as uncharged tRNAs remain stuck and block de novo protein synthesis. As proteins including Dph1 and Dph2 lack replenishment after cycloheximide treatment, their stability versus degradation can be readily traced over time in Western blots (Figure 4B). We grew WT (*DPH1-HA DPH2-c-Myc*) and mutant (*dph1C368-HA DPH2-c-Myc*) yeast strains to the exponential phase before applying cycloheximide (100 μg/mL) for up to nine hours (Figure 4B and Figure S8). Total extracts from the resulting fractions were subjected to Western blots using anti-HA and anti-c-Myc antibodies to detect either subunit of Dph1•Dph2 (Figure 4B). While we observed no change in Dph1-HA (anti-HA) or Dph2-c-Myc (anti-c-Myc) over nine hours from WT cells, both subunits of the heterodimer from diphthamide mutant *dph1C368S* vanished unanimously after three hours into the cycloheximide chase (Figure 4B). Similarly, we examined Dph1•Dph2 instability upon chasing the *dph2* double Cys-to-Ser variant (*C106*,*107S-c-Myc DPH1-HA*) using cycloheximide. Although Dph1•Dph2 levels decreased over time as a result of the combined (*C106*,*107S*) substitutions in the TCM of Dph2 (Figures S9 and S10), Dph1-HA and Dph2-c-Myc protein instability appeared significantly less prominent compared to the critical *dph1* mutant above (Figure 4B). Thus, after three hours into the cycloheximide chase, relative stable subunit pools amounted to 69% (Dph1-HA) and 55% (Dph2-c-Myc) (Figures S9 and S10). In sum, Dph1•Dph2 instability is enhanced in either background, and subunit degradation that associates with Cys-to-Ser substitutions is predominantly seen and prone to alterations in the Fe-S-cluster motif from Dph1, which likely is site-differentiated for SAM binding and cleavage via the Dph1•Dph2 enzyme [9,10,21,42].

## 4. Conclusions and Perspectives

In this study, we further analyzed atypical radical SAM and Fe-S motifs of Dph1•Dph2, a non-canonical RS enzyme from yeast. We focused primarily on hitherto ill-defined cysteines in Dph1 (Cys-134) and Dph2 (Cys-106) that form TCMs conserved in eukaryotic members of the Dph1•Dph2 heterodimer (Figure 1). We were able to rule out functional relevance for Dph1Cys-134 in the diphthamide-modification pathway. However, we found that a loss of Dph2Cys-107 (a mild enzyme defect when substituted alone for Ser) together with Dph2Cys-106 (also a weak diphthamide defect on its own) renders yeast cells almost unable to synthesize diphthamide on eEF2. Congruently, eEF2 from the double mutant (*C106*,*107S*) accumulates unmodified eEF2 (Figure 2) that hardly has acceptor activity for ADP-ribosylation, a diphthamide-dependent modification reaction (Figure 3). Previously, Dong et al. (2019) identified Fe-S cluster binding roles for Cys-107, Cys-128, and Cys-362 in the Dph2 subunit and suggested for Cys-106 a potential fourth ligand role [9]. Our data presented are in strong support of this option, and we show that Cys-106 and Cys-107 indeed functionally overlap in the TCM of Dph2 and contribute to full Dph1•Dph2 enzyme activity. In line with cooperativity, the double mutant (*C106*,*107S*) triggers phenotypes and properties that add up in relation to each single mutant (*C106S* or *C107S*), indicating a bone fide diphthamide defect in vivo (Figure 2 and Figure 3).

To further put our data into perspective, we revisited the structural models of the cofactor binding motifs in the Dph1•Dph2 heterodimer (Figure 1). We compared the distance between iron ions and proximal cysteines in the TCMs of Dph1 (Cys-133: 1.5 Å, essential residue; Cys-134: 3.9 Å, non-essential residue) (Figure S11) in comparison to the TCM in Dph2 (cooperating residues Cys-106: 2.0 Å and Cys-107: 3.0 Å). Obviously, the latter two share closer distances towards the nearest iron ion in the Fe-S cluster model, which supports our data, reinforcing a functional overlap between Cys-106 and Cys-107 in Dph2 (but not Cys-133 and Cys-134 in Dph1) (Figure S11). Other microbial Fe-S clusters contained in proteins like NuoB ligate two irons of the cofactor with two tandem cysteines [45,46]. In case of Dph2, such a modified stoichiometry (CCX_21_CX_234_C) may update and expand the Fe-S cluster ligands to Cys-106, Cys-107, Cys128, and Cys-362 (Figure 1B). If this is the case, the cofactor not necessarily exposes a unique, Cys-free iron typical of site-differentiated radical SAM motifs required for the binding and cleavage of the cosubstrate SAM [1,2,3,4]. This notion is in line with recent genetic and biochemical evidence [9,10,42], showing that subunit Dph1 (but not Dph2) contains a functional SAM pocket in the Dph1•Dph2 dimer. Whether other combined Dph2 mutations (for instance, *C106,362S* or *C107,362S*) would trigger phenotypes reminiscent of our characterized *C106,107S* mutant is an an interesting question to address. If this turned out to be the case, this could support the hypothesis that more substantial changes (such as combined cysteine replacements) are required to affect Dph2 (rather than Dph1) function and stability. Irrespective of these novel insights, further studies will be required to elucidate Fe-S cluster function in the Dph2 subunit of the asymmetric RS enzyme. Previous evidence suggested it is necessary for Dph1•Dph2 activation by the iron-binding and electron carrier protein Dph3 (aka Kti11) [47,48,49,50], which in complex with Dph8 (aka Kti13) also acts on Elongator, another translation-relevant RS enzyme complex that modifies tRNA anticodons [51,52,53]. However, activation details remain unclear to this end.

Nonetheless, our data identified cysteines in the TCM of Dph2 (Cys-106 and Cys-107) that cooperate in Dph1•Dph2 functionality and found that Cys-133 in the TCM of Dph1 is particular critical for the structural integrity of the dimeric RS enzyme. Likewise, other Cys residues in the non-canonical radical SAM motif of Dph1 (Cys-239; Cys-368) ensure Dph1•Dph2 stability, suggesting both cofactor/cluster motifs in the Dph1 and Dph2 subunits communicate on the level of enzyme integrity and stability. Thus, as with viperin, and other medically relevant classical RS enzyme, the structural integrity of the non-canonical RS enzyme Dph1•Dph2 also relies on functional binding sites for SAM and Fe-S cofactors [4,6,54]. Intriguingly, clinically relevant human DPH1•DPH2 variants include a Tyr replacement in DPH2Cys-342, which in yeast corresponds to Dph2Cys-362 and was identified to be inactive upon Tyr substitution [21,22], likely through Fe-S cluster loss and enhanced protein instability. Thus, insights into the consequences of Cys-based cofactor motif substitutions as reported can further address the critical roles diphthamide modification on eEF2 plays for accurate mRNA translation and protein synthesis in eukaryotes [55,56,57,58] and, importantly, help understand the molecular causes of pathogenic mutants for the diagnosis of patients with DDS symptoms [18,19,20,21,22]. Our identification of functionally and structurally important Dph1•Dph2 cysteines can directly be linked to diphthamide synthesis on eEF2 and thus is likely to impact on mRNA-translation elongation and cellular physiology with clinical relevance. Last but not least, together with the recent discovery of another non-canonical RS enzyme that uses ACP rather than classical dAdo radicals for arsinothricin biosynthesis [59], our report reinforces both the diversity and emerging plasticity of radical SAM family members.

## Data Availability

All the data can be found in the manuscript and Supplementary Materials.

## Supplementary Materials

The following supporting information can be downloaded at https://www.mdpi.com/article/10.3390/biom14040470/s1. Table S1: Yeast strains used and generated in this study. Table S2: Primers used for PCR-based gene engineering and genomic verification. Figure S1: Alignment between archaeal *Ph*Dph2 and eukaryal Dph1 and Dph2 sequences. Figure S2: Original Western blot images underlying parts of the data presented in Figure 3A. Figure S3: Further original Western blot images underlying parts of the data presented in Figure 3A. Figure S4: Original Western blot images underlying parts of the data presented in Figure 3B. Figure S5: Original Western blot images underlying the data presented in Figure 4A. Figure S6: Substitutions of functionally important cysteines in Dph2 result in decreased amounts of both subunits of the Dph1•Dph2 dimer. Figure S7: Original Western blot images underlying the data presented in Figure S5. Figure S8: Original Western blot images underlying the data presented in Figure 4B. Figure S9: Cycloheximide chase of *dph2C106,107S* reveals accelerated Dph1•Dph2 decay. Figure S10: Original Western blot images underlying the data presented in Figure S9. Figure S11: Structural modelling highlights conserved cysteines in radical SAM and Fe-S motifs of Dph1•Dph2.
