# Supplementary material for: Functional Integrity of Radical SAM Enzyme Dph1•Dph2 Requires Non-Canonical Cofactor Motifs with Tandem Cysteines
Source: Biomolecules. 2024 Apr 11;14(4):470. doi: 10.3390/biom14040470 (PMC11048331; doi:10.3390/biom14040470)
Supplement: Supplementary file 1 [file biomolecules-14-00470-s001.zip › biomolecules-2955549-supplementary.pdf]

biomolecules

Supplementary Materials

Communication

Functional integrity of radical SAM enzyme Dph1•Dph2 requires non-canonical cofactor motifs with tandem cysteines

Koray Ütkür [1], Klaus Mayer [2], Shihui Liu [3], Ulrich Brinkmann [2] and Raffael Schaffrath [1,*]

[1] Institut für Biologie, Fachgebiet Mikrobiologie, Universität Kassel, Kassel, Germany
[2] Roche Pharma Research and Early Development (pRED), Large Molecule Research, Roche Innovation Center Munich, Penzberg, Germany
[3] Division of Infectious Diseases, Department of Medicine, University of Pittsburgh School of Medicine, Pittsburgh, PA, USA

* Correspondence: schaffrath@uni-kassel.de

1. Supplementary Tables

Table S1. Yeast strains used and generated in this study.

Strain	Genotype	Source
BY4741	*MAT***a** *his3Δ1 leu2Δ0 met15Δ0 ura3Δ0*	Euroscarf *
Y02262	BY4741 *dph1Δ::kanMX4*	Euroscarf
Y05041	BY4741 *dph2Δ::kanMX4*	Euroscarf
KU28	BY4741 *DPH1-(HA)$_6$::HIS3MX6; DPH2-(c-Myc)$_3$::kanMX*	This study
KU7	BY4741 *dph1C133S; KlLEU2*	This study
KU114	BY4741 *dph1C133S-(HA)$_6$::HIS3MX6; KlLEU2; DPH2-(c-Myc)$_3$::kanMX*	This study
KU95	BY4741 *dph1C134S; KlLEU2*	This study
KU115	BY4741 *dph1C134S-(HA)$_6$::HIS3MX6; KlLEU2; DPH2-(c-Myc)$_3$::kanMX*	This study
KU96	BY4741 *dph1C133,134S; KlLEU2*	This study
KU116	BY4741 *dph1C133,134S-(HA)$_6$::HIS3MX6; KlLEU2; DPH2-(c-Myc)$_3$::kanMX*	This study
KU8	BY4741 *dph1C239S; KlLEU2*	This study
KU31	BY4741 *dph1C239S-(HA)$_6$::HIS3MX6; KlLEU2; DPH2-(c-Myc)$_3$::kanMX*	This study
KU9	BY4741 *dph1C368S; KlLEU2*	This study
KU32	BY4741 *dph1C368S-(HA)$_6$::HIS3MX6; KlLEU2; DPH2-(c-Myc)$_3$::kanMX*	[1]
KU14	BY4741 *dph2C106S; KlLEU2*	This study
KU265	BY4741 *DPH1-(HA)$_6$::HIS3MX6; dph2C106S-(c-Myc)$_3$::kanMX*	This study
KU16	BY4741 *dph2C107S; KlLEU2*	This study
KU112	BY4741 *DPH1-(HA)$_6$::HIS3MX6; dph2C106,107S-(c-Myc)$_3$::kanMX*	This study
KU15	BY4741 *dph2C106,107S; KlLEU2*	This study
KU111	BY4741 *DPH1-(HA)$_6$::HIS3MX6; dph2C106,107-(c-Myc)$_3$::kanMX*	This study
KU17	BY4741 *dph2C362S; KlLEU2*	This study
KU113	BY4741 *DPH1-(HA)$_6$::HIS3MX6; dph2C362S-(c-Myc)$_3$::kanMX*	This study

* http://www.euroscarf.de/index.php?name=News

Table S2. Primers used for PCR-based gene engineering and genomic verification.

Name	Sequence (5′ → 3′)	Usage **
DPH1KOURAF	CTCATGAACTATCTGCTGCGAATTTTAAGGATAATCGGATAGCC AGCTGAAGCTTCGTACGC	ko
DPH13'UTRLEUF	CGTTTTTGACGGCTTGCAGGCGAAACTAAATTGTCTAAAATTCA AAACCAGCTGAAGCTTCGTACG	smi
DPH13'UTRLEUR	GAATAAAAATAGGCTTGACCAGCAGTGATATCAAGTTAGAAGG CATTGCATAGGCCACTAGTGGATCTG	ko/smi
DPH15'UTRF	GTGATGGTAGATTATAGCAAG	ko-ver
DPH13'UTRR	GGAAATATGCTTGGCAAACTC	ko-ver
DPH2KOURAF	AAAGAGTTAAGATGATTAGTGATGGATTTCTAAGTGGCAGCGTTGC AGCTGAAGCTTCGTACGC	ko
DPH23'UTRHISF	GCCTTGGAAATTAGCCGCCAAAATGGGATATACATTCCGTGCGAAC AGCTGAAGCTTCGTACGC	smi
DPH23'UTRHISR	AAACTAGTGATTTTTAAGATGATACCCGGCCTTCCACGCGGTCACG CATAGGCCACTAGTGGATCTG	smi
DPH25'UTRF	GTTTTAATGCTATGGTAGACTTCAG	ko-ver
DPH23'UTRR	GCACAAAGCACCTTTATTGC	ko-ver
DPH1C133SFW	GATGTGTCTTATGGTGCATCCTGTATTGATGA	sdm
DPH1C133SRV	CTAGCAGTAAAATCATCAATACAGGATGCACCA	sdm
DPH1C134SFW	GATGTGTCTTATGGTGCATGCTCTATTGATGA	sdm
DPH1C134SRV	CTAGCAGTAAAATCATCAATAGAGCATGCACCA	sdm
DPH1C133S/C134SFW	GATGTGTCTTATGGTGCATCCTCTATTGATGA	sdm
DPH1C133S/C134SRV	CTAGCAGTAAAATCATCAATAGAGGATGCACCA	sdm
DPH1C239SFW	CCTCTATCGAGGGGTGAAGTATTGGGGTCTACTTCTGAAAG	sdm
DPH1C239SRV	GTATTGTTCTTTATCTAATCTTTCAGAAGTAGACCCCAATA	sdm
DPH1C368SFW	CAAATTGATGTTTTTGTTCAGGTCGCATCTCCTAGACTGTCC	sdm
DPH1C368SRV	GAAGGCATAACCCCAATCGATGGACAGTCTAGGAGATGCGACCTG	sdm
DPH2C106SFW	CTGACACAGCGTACAGTGCATCCTGTGTAGACG	sdm
DPH2C106SRV	CGTGTTCAGCAGCGACCTCGTCTACACAGGATGCAC	sdm
DPH2C107SFW	CTGACACAGCGTACAGTGCATGCTCTGTAGACG	sdm
DPH2C107SRV	CGTGTTCAGCAGCGACCTCGTCTACAGAGCATGCAC	sdm
DPH2C106S/C107SFW	CTGACACAGCGTACAGTGCATCCTCTGTAGACG	sdm
DPH2C106S/C107SRV	CGTGTTCAGCAGCGACCTCGTCTACAGAGGATGCAC	sdm
DPH2C362SFW	GATATTTGGTGCATTCTCGGTTCTAGCCAAAG	sdm
DPH2C362SRV	CAACGATGATACCGCTTTGGCTAGAACCGAG	sdm
DPH1S2	CATATGTAACAGGAAGACAAGTGACAACAAAAACTATTTAAAC TAATCGATGAATTCGAGCTCG	tag
DPH1S3	CGAAGCTAAAGGATACGGGCGTGGGAAACTCCGAAACATGC GATTGAACGTACGCTGCAGGTCGAC	tag
DPH2S2	CTTGGATTTAAATAGAGAAGTCGAGGGAAACAAATTATAAGAGTC AATCGATGAATTCGAGCTCG	tag
DPH2S2	CGTGGTTATGGATTTGATCGCGAAGACGCTATGAAAAAGGAAAAC AAACGTACGCTGCAGGTCGAC	tag

** Abbreviations used:

(i) ko – gene knock-out; (ii) smi – selection marker insertion; (iii) ko-ver – verification of gene knockout; (iv) sdm – site-directed mutagenesis; (v) tag – epitope tagging

biomolecules

2. Supplementary Figures

Figure S1

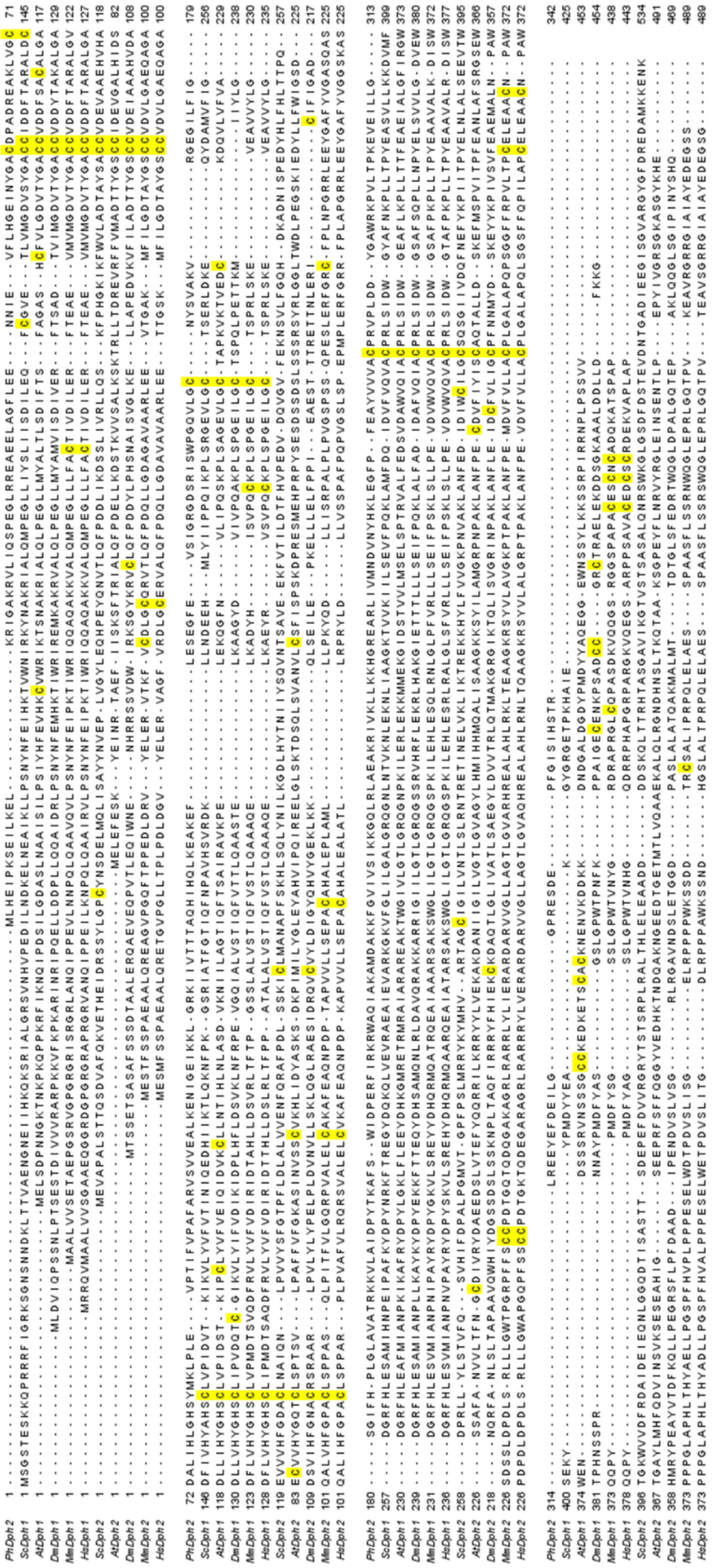

Figure S1. Alignment between archaeal *Ph*Dph2 and eukaryal Dph1 and Dph2 sequences. Amino acid sequences of *Ph*Dph2 were aligned to sequences of Dph1 and Dph2 from *S. cerevisiae*, *A. thaliana*, *D. melanogaster*, *M. musculus* and *H. sapiens*. All cysteines are marked in yellow. For detailed Cys-based cofactor motifs and TCMs, see Figure 1.

biomolecules

Figure S2

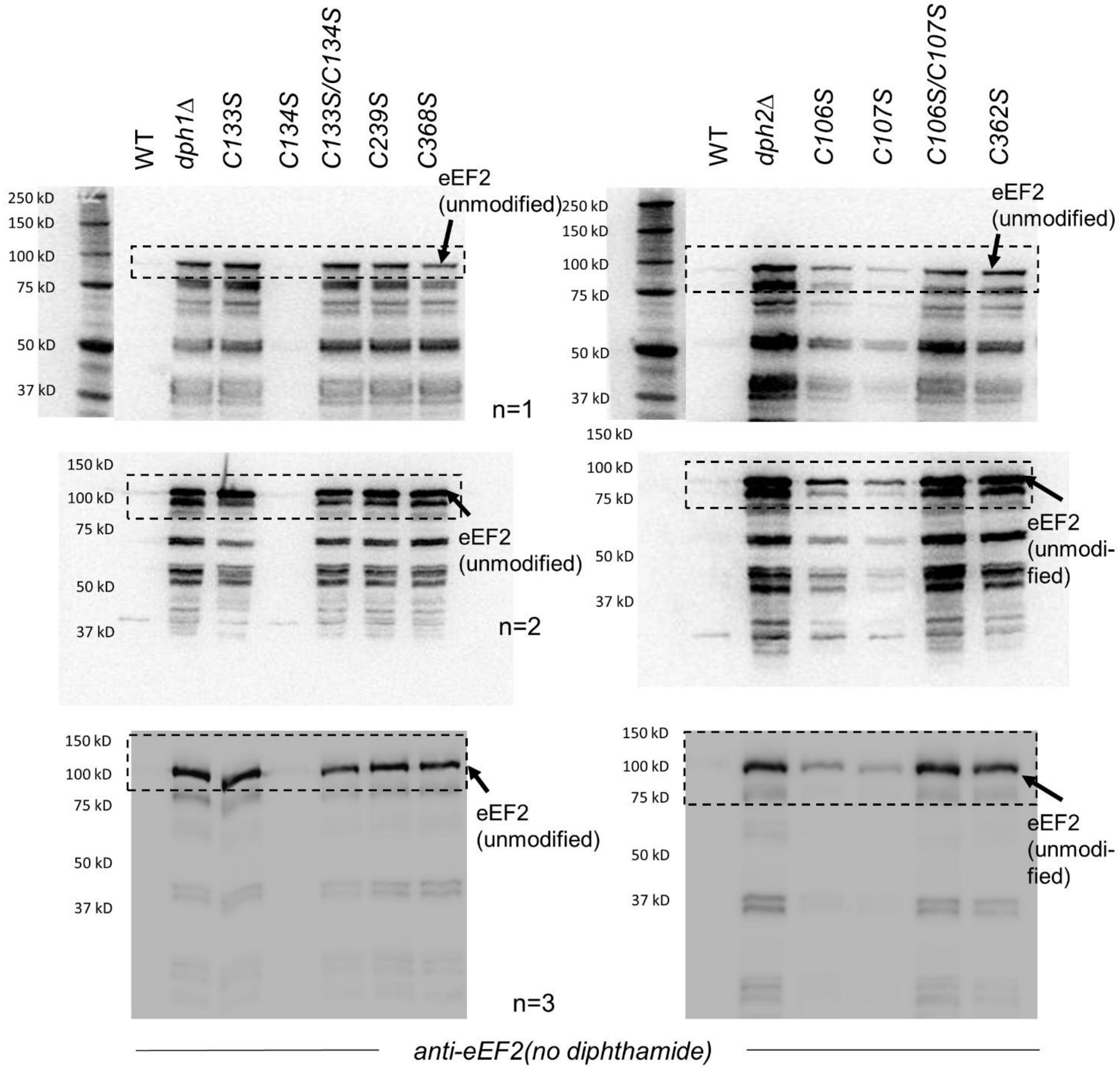

Figure S2. Original Western blot images underlying parts of the data presented in Figure 3A (indicated by the areas of the dotted boxes). Band signal intensities were used for densitometric and statistical analyses.

Figure S3

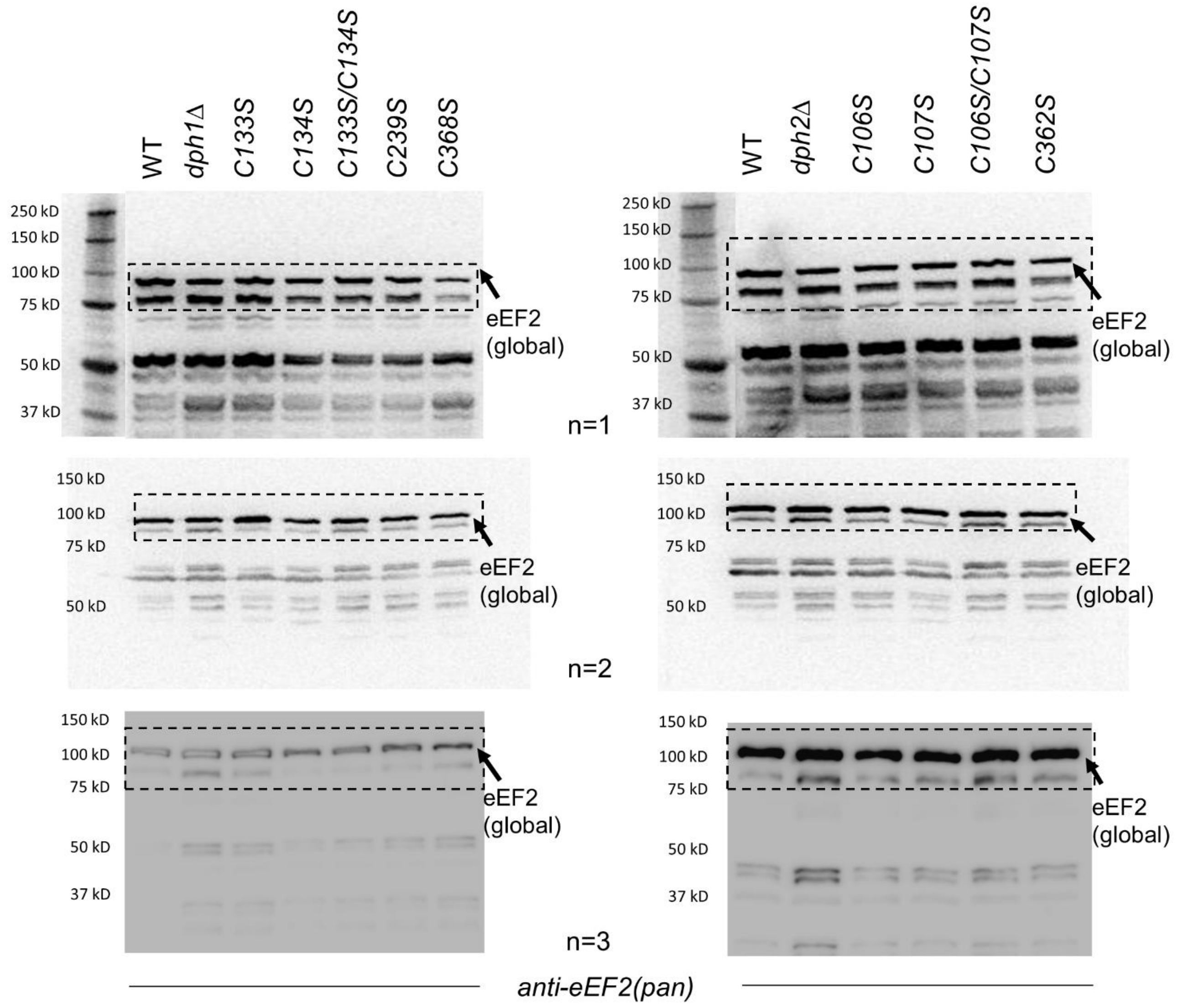

Figure S3. Further original Western blot images underlying parts of the data presented in Figure 3A (indicated by the areas of the dotted boxes). Band signal intensities were used for densitometric and statistical analyses.

biomolecules

Figure S4

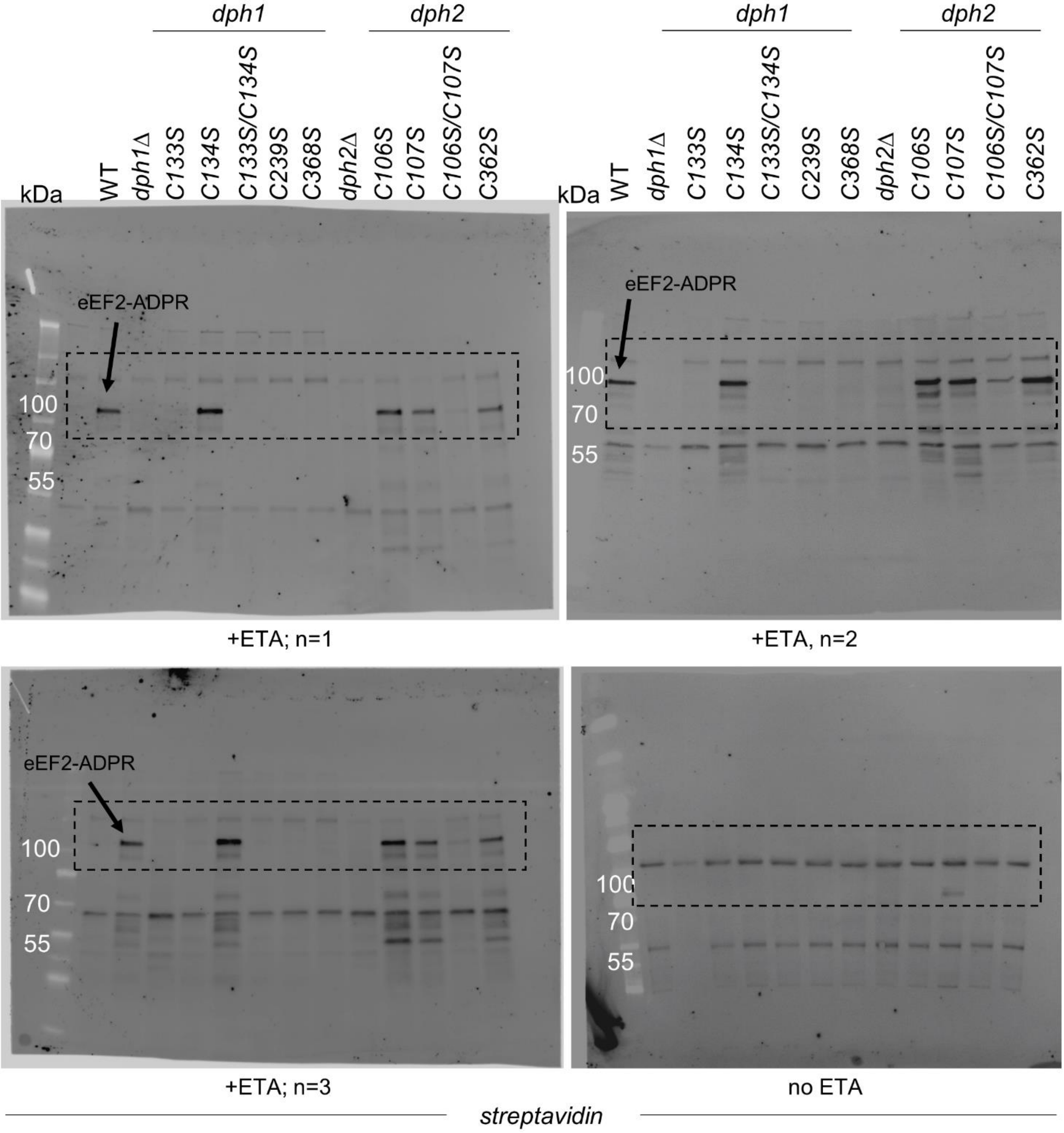

Figure S4. Original Western blot images underlying parts of the data presented in Figure 3B (indicated by the areas of the dotted boxes). Band signal intensities were used for densitometric and statistical analyses.

Figure S5

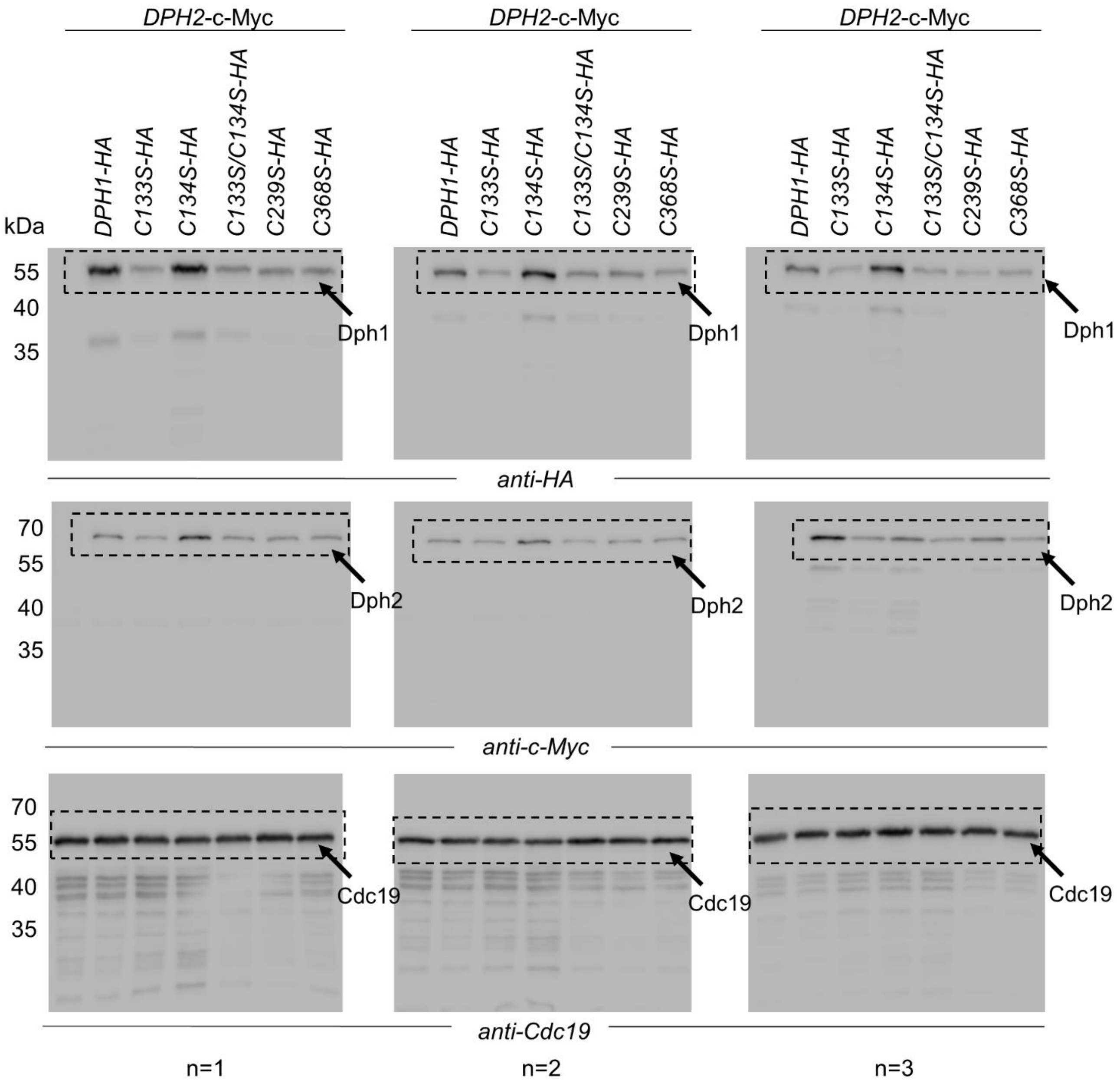

Figure S5. Original Western blot images underlying the data presented in Figure 4A (indicated by the areas of the dotted boxes). Band signal intensities were used for densitometrical and statistical analyses

Figure S6

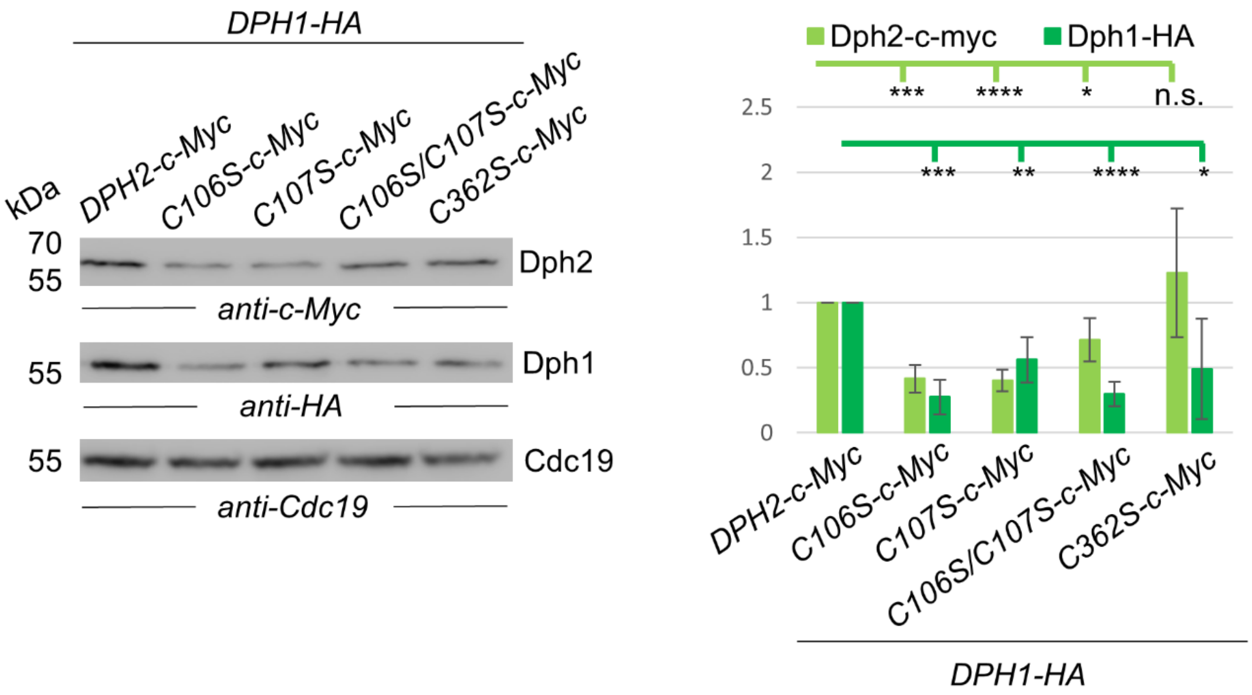

Figure S6. Substitutions of functionally important cysteines in Dph2 result in decreased amounts of both subunits of the Dph1•Dph2 dimer. Western blot analyses of *DPH2* mutants were conducted to detect cellular pools of Dph1-HA (*anti-HA*) and Dph2-c-Myc (*anti-c-Myc*). Detection of the yeast pyruvate kinase Cdc19 (*anti-Cdc19*) served as control for sample loading. Technical repetitions (n=5) were followed by densitometric quantification of signal intensities and standard t-test for statistical analyses * = p<0.05; ** = p<0.01; *** = p<0.001; **** = p<0.0001; n.s. = not significant. For original Western blot images, see also Figure S7.

Figure S7

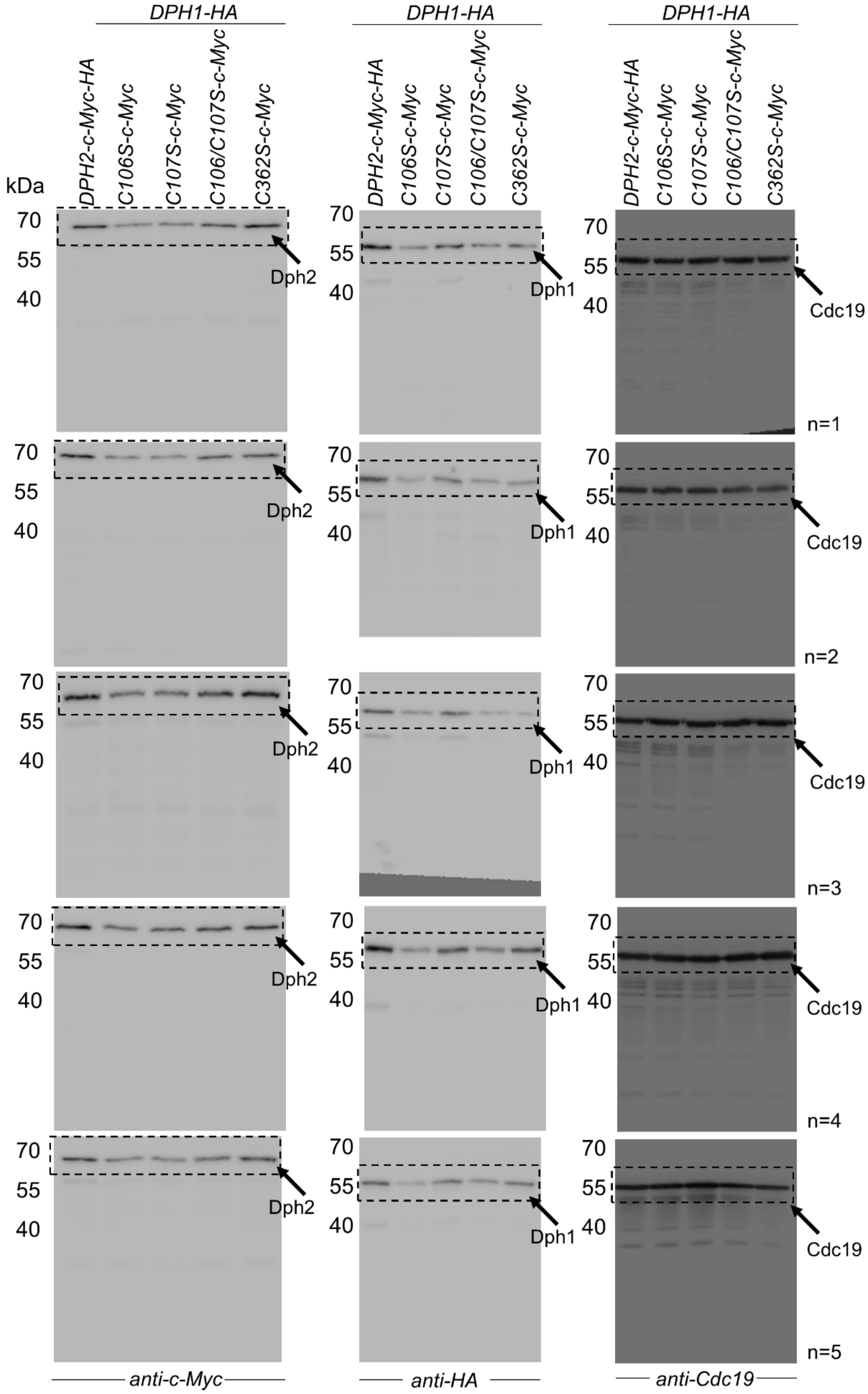

Figure S7. Original Western blot images underlying the data presented in Figure S6 (indicated by the areas of the dotted boxes). Band signal intensities were used for densitometric and statistical analyses.

Figure S8

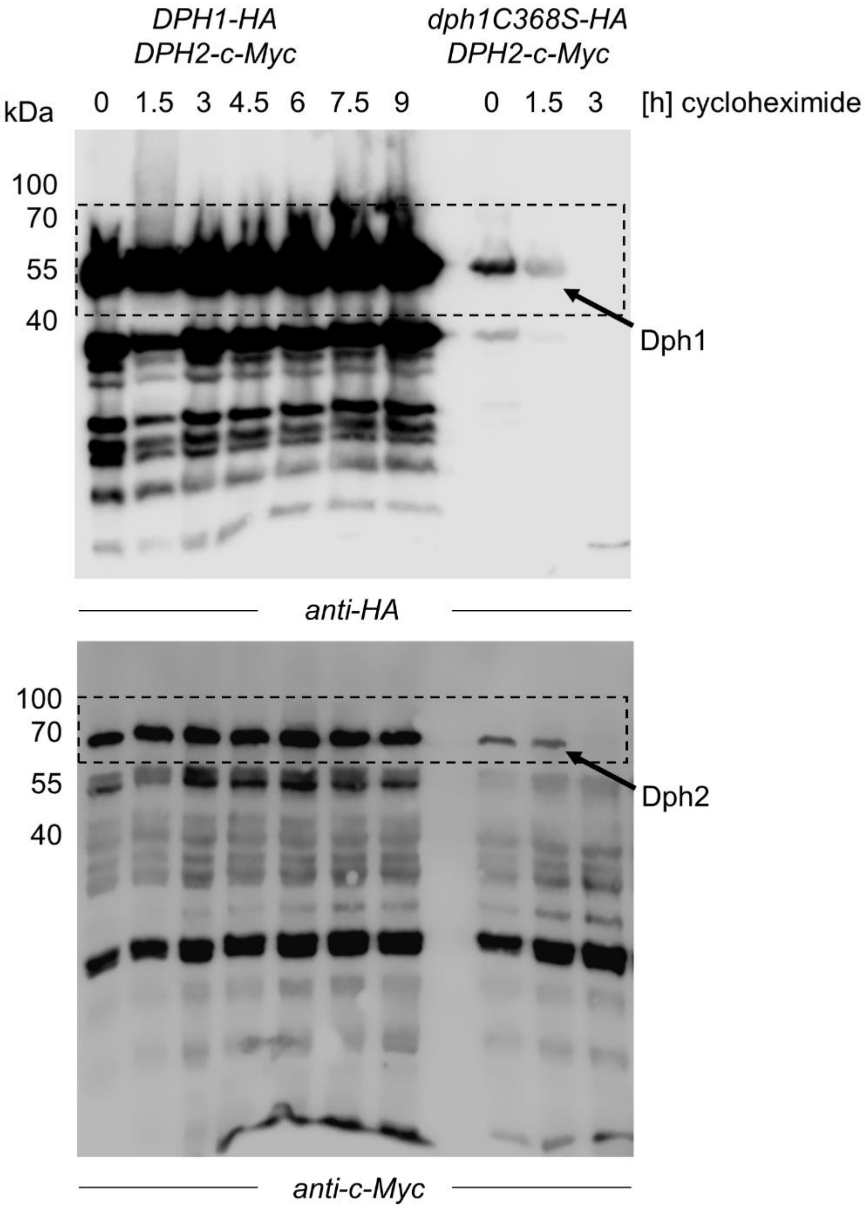

Figure S8. Original Western blot images underlying the data presented in Figure 4B (indicated by the areas of the dotted boxes).

Figure S9

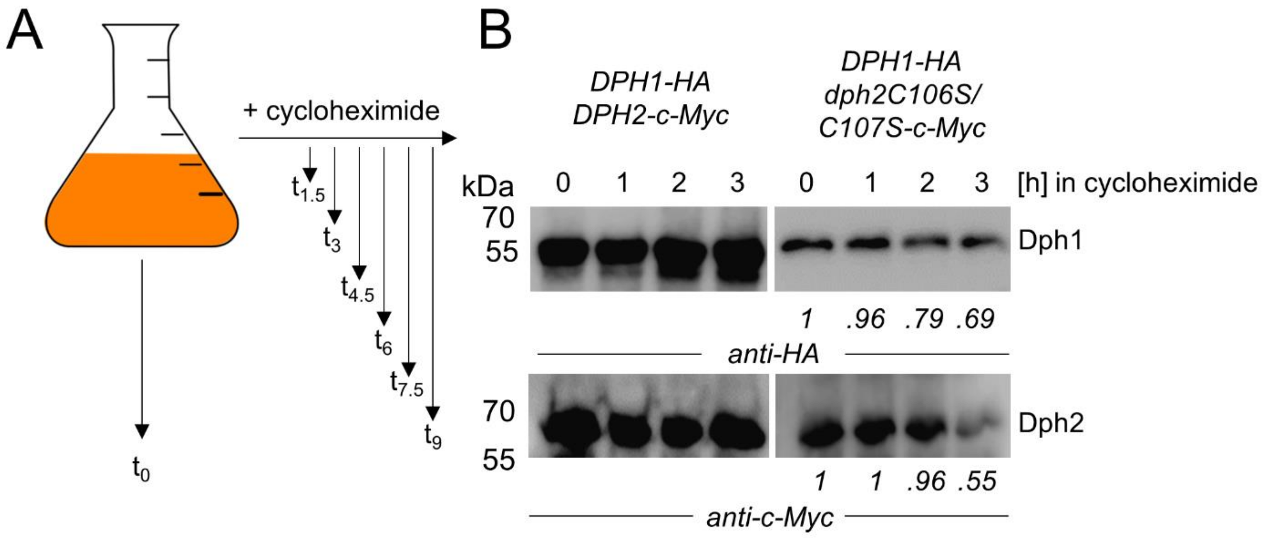

Figure S9. Cycloheximide chase of *dph2C106,107S* reveals accelerated Dph1•Dph2 decay. (**A**) Schematic workflow of the cycloheximide chase. Yeast cells coding for C-terminally tagged versions of Dph1 and Dph2 were grown to reach the exponential phase (t_0) before 100µg/mL cycloheximide were added to the cultures. Samples were taken at indicated time points before (t0) and after cycloheximide addition for total protein extraction and (**B**) Western blot analysis. Dph1 and Dph2 band signal intensities from the *dph2* mutant were densitometrically analysed and normalised to t_0. For further Western blot images, see Figure S10.

biomolecules

Figure S10

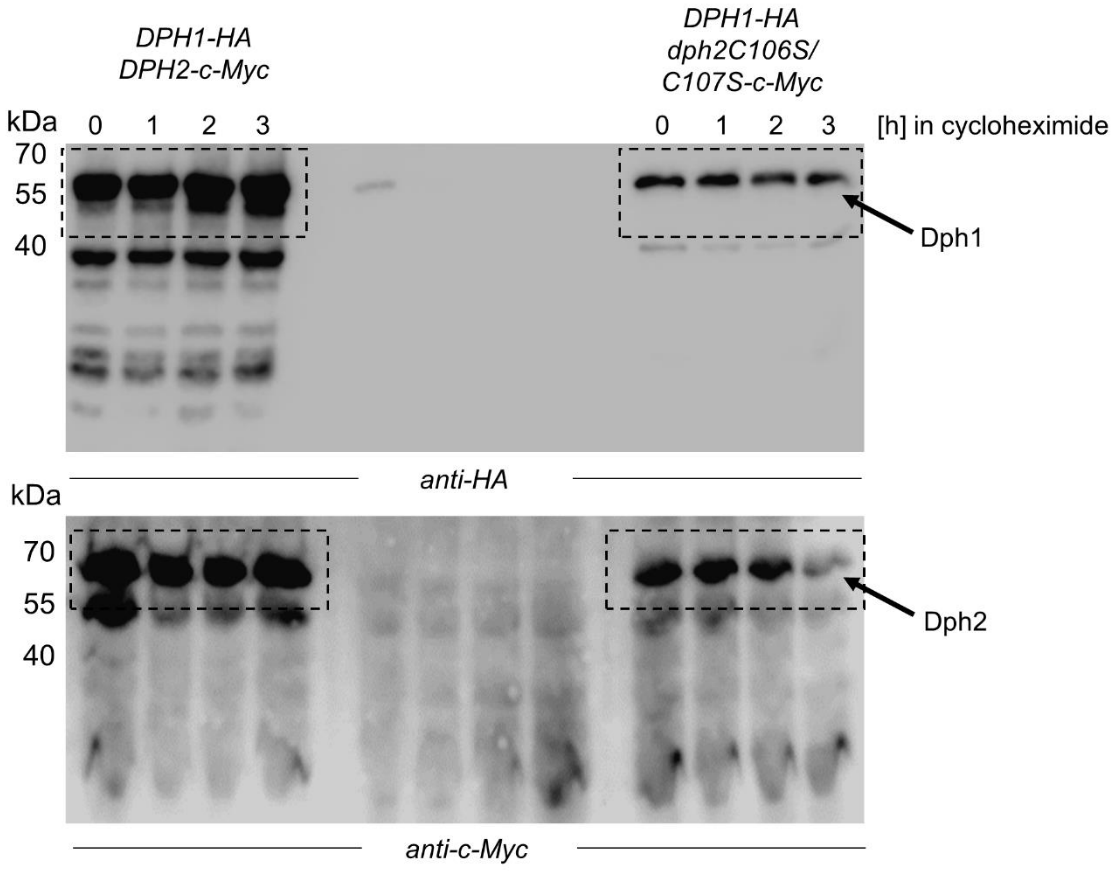

Figure S10. Original Western blot images underlying the data presented in Figure S9 (indicated by the areas of the dotted boxes). Band signal intensities were used for densitometric analyses.

Figure S11

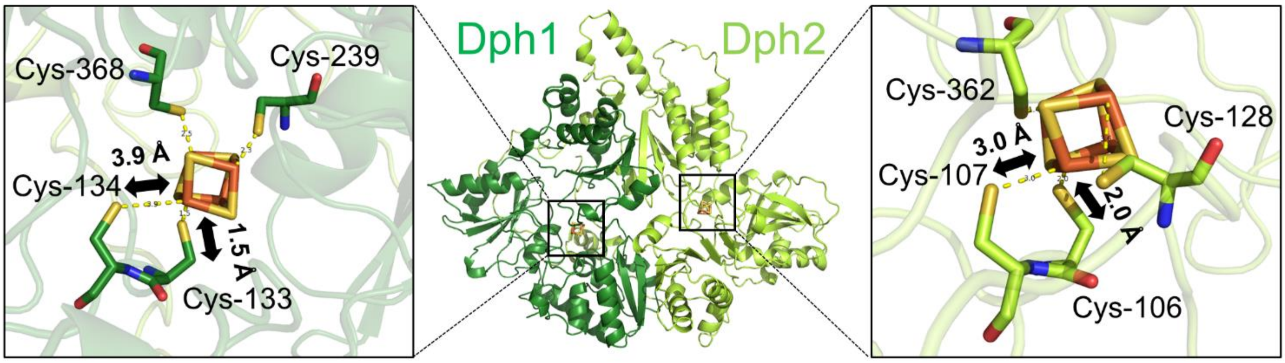

Figure S11. Structural modelling highlights conserved cysteines in radical SAM and Fe-S motifs of Dph1•Dph2. Center: Amino acid sequences of *S. cerevisiae* Dph1 (green-forest) and Dph2 (green-limon) were modeled as a heterodimer with AlphaFold/ColabFold [2,3]. Given Dph1•Dph2 model was then structurally aligned with PDB: 6bxn (not shown) [4] and illsutrated in its cartoon structure with PyMOL version 1.3. Left: A zoom into the active center of Dph1 shows a [4Fe-4S] cluster with three conserved FeS ligands, Cys-133, Cys-239 and Cys-368, illustrated as sticks. Distances of conserved cysteines to the next iron ion (1.5 A, 2.3A and 2.5 A respectively) are labeled according to the yellow dotted lines. In addition, Cys-134 follows Cys-133 in close proximity to the next iron ion in 3.9 A distance. Right: A zoom into the active center of Dph2 show a [Fe_4-S_4] and conserved cofactor binding cysteines 107, 128 and 362 shown as sticks. Distances of conserved cysteines to the next iron ion (3.0 A, 1.4 A and 3.6 A respectively) are labeled according to the yellow dotted lines. In addition, Cys-106 adjacent to Cys-107 is in close proximity to the next iron ion by 2.0 A distance.

3. Supplementary References

1. Ütkür, K.; Schmidt, S.; Mayer, K.; Klassen, R.; Brinkmann, U.; Schaffrath, R. *DPH1* gene mutations identify a candidate SAM pocket in radical enzyme Dph1•Dph2 for diphthamide synthesis on EF2. *Biomolecules* **2023**, *13*, 1655, doi: 10.3390/biom13111655.
2. Jumper, J.; Evans, R.; Pritzel, A.; Green, T.; Figurnov, M.; Ronneberger, O.; Tunyasuvunakool, K.; Bates, R.; Žídek, A.; Potapenko, A.; et al. Highly accurate protein structure prediction with AlphaFold. *Nature* **2021**, *596*, 583–589, doi: 10.1038/s41586-021-03819-2.
3. Mirdita, M.; Schütze, K.; Moriwaki, Y.; Heo, L.; Ovchinnikov, S.; Steinegger, M. ColabFold: Making protein folding accessible to all. *Nat. Methods* **2022**, *19*, 679–682, doi: 10.1038/s41592-022-01488-1.
4. Dong, M.; Kathiresan, V.; Fenwick, M.K.; Torelli, A.T.; Zhang, Y.; Caranto, J.D.; Dzikovski, B.; Sharma, A.; Lancaster, K.M.; Freed, J.H.; et al. Organometallic and radical intermediates reveal mechanism of diphthamide biosynthesis. *Science* **2018**, *359*, 1247–1250, doi: 10.1126/science.aao6595.
